# Trends of repeated emergency department visits among adolescents and young adults for substance use: A repeated cross-sectional study

**DOI:** 10.1371/journal.pone.0282056

**Published:** 2023-02-22

**Authors:** Soyeon Kim, John Weekes, Matthew M. Young, Nicole Adams, Nathan J. Kolla

**Affiliations:** 1 Waypoint Centre for Mental Health Care, Penetanguishene, Ontario, Canada; 2 McMaster University, Hamilton, Ontario, Canada; 3 Carleton University, Ottawa, Ontario, Canada; 4 Canadian Centre on Substance Use and Addiction, Ottawa, Ontario, Canada; 5 Greo, Ottawa, Canada; 6 Centre for Addiction and Mental Health, Toronto, Ontario, Canada; Massachusetts General Hospital and Harvard Medical School, UNITED STATES

## Abstract

Emergency Department (ED) visits for substance-related concerns among young people have been increasing in recent years. Understanding the factors related to repeated ED visits (two or more ED visits per year) for substance use concerns among young people is critical to developing a more efficient mental healthcare system that does not overburden ED and that provides efficient care for substance use patients. This study examined trends of substance use-related ED visits and factors related to repeated ED visits (two or more ED visits per year, in comparison to one ED visit per year) among adolescents and young adults (aged 13 to 25 years) in the province of Ontario, Canada. Binary logistic regression models were conducted to examine associations between hospital-related factors (hospital size, urbanicity, triage level, ED wait time) and visit status (2+ vs 1 ED visit/year), controlling for patient characteristics (age/sex). A population-based, repeated cross-sectional data over a 10-year period (2008, 2013, and 2018) was used. The proportion of substance use-related repeated ED visits significantly and consistently increased in the year 2013 and 2018 compared to 2008 (2008 = 12.52%, 2013 = 19.47%, 2018 = 20.19%). Young adult, male, medium-sized hospital, urban location, wait times longer than 6 hours, and symptom severity was associated with increased numbers of repeated ED visits. Furthermore, polysubstance use, opioid use, cocaine use, and stimulant use were strongly associated with repeated ED visits compared with the use of substances such as cannabis, alcohol and sedatives. Current findings suggest that repeated ED visits for substance use concerns could be reduced by policies that reinforce evenly distributed mental health and addiction treatment services across the provinces in rural areas and small hospitals. These services should put special efforts into developing specific (e.g., withdrawal/treatment) programming for substance-related repeated ED patients. The services should target young people using multiple psychoactive substances, stimulants and cocaine.

## Introduction

Globally, mental health and addiction challenges are the leading cause of disability among young people [1]. Despite the gravity of mental health and addiction problems, approximately 1 in 5 individuals receive the services and support needed to help manage these challenges [2, 3]. For instance, access to community mental health and addiction resources has decreased over the past few decades (i.e., a decline in qualified psychiatrists and other service providers) [4–6]. Consequently, the emergency department (ED) has become the fastest-growing source of assistance for mental health and addiction challenges in Canada [3, 7–10]. In Canada, the rate of ED visits for mental health and addiction concerns has substantially increased from 2009 to 2017 [6]. Despite this increasing trend, Ontario’s acute hospital bed capacity has remained constant over the past two decades [11], disproportionate to the population increase in the province [12]. An over-reliance on EDs for mental health and addiction challenges may indicate an inefficient mental healthcare system. Accordingly, informing evidence-based factors related to ED visits is critical.

Increased demand for ED for mental health and addiction services should involve increased targeted resources [1, 13, 14] and efficient transition to outpatient mental health care [15]. However, approximately 60% of ED patients are not followed up by outpatient care in Canada [6, 15]. Further, one in three patients who visit the ED for mental health and addiction issues do not have prior records of outpatient service utilization [6]. Also, a high rate (22%) of ED patients return to the ED for mental health and addiction-related issues within six months [14]. Overall, despite the increased demand for mental health and addiction assistance, the healthcare system’s overall efficiency has not improved over time accordingly [6, 16]. The current mental health and addiction outpatient service utilization trend may signal Canada’s chronic and acute unmet mental health and addiction treatment needs [17].

Understanding factors related to increased ED visits is critical to inform policy strategies to enhance the mental health and addiction (MHA) healthcare system. In Canada, ED visits for MHA increased by 89.1% between 2006 and 2017, with the greatest rise observed for adolescents and young adults [6, 16, 18]. Also, repeat visits represented a significant proportion of the increased visits among youth [13, 14]. For instance, 39% to 45% of the adolescents and young adults visiting the ED for MHA concerns returned to ED for the same concerns [13, 14]. Lastly, substance use-related ED visits showed the highest increase between 2009 and 2017 [6]. In sum, adolescent and young adult age groups, substance use-related challenges, and repeated visits are possible contributing factors for increased ED visits.

Previous studies indicate that 37% of alcohol-related ED patients re-visited the ED at least once [19]. Individuals who visited the ED in Quebec for alcohol-related presentations were more likely to be male, with a median age of 20 years [20]. 68% of the alcohol-related ED visitors showed an altered consciousness level, and 23% presented a life-threatening state [20]. Alcohol is not the only substance that brings adolescents and young adults to the ED. In October 2018, the Government of Canada legalized non-medical cannabis use. In the US, cannabis-related ED visits increased between 2004 and 2011 [21, 22]. Indeed, the 12-17-year old age group was more likely to visit the ED for cannabis use and evidenced the largest increase in ED visits between 2004 and 2011[21]. Furthermore, Canada is the second-largest consumer of prescription opioids in the world after the US, with approximately 4,000 deaths due to opioid poisoning in 2017 alone [23]. Youth’s opioid-related ED visits have increased by almost 50% in Ontario between 2012–2013 and 2016–2017 [24].

Reflecting these trends, Canada’s federal, provincial and territorial governments endorsed agreements in August 2017 to focus on the shared health priorities of home, community, and mental health and addiction care [25, 26]. Particularly, the Ontario Ministry of Health and Long-Term Care (MOHLTC) has made significant investments in a provincial strategy focused on improving mental health and addiction services, with a particular emphasis on "right care, right time, right place," allowing access to timely and appropriate services in the community when mental health and addiction issues arise [27].

The present study aims to inform evidence-based factors for repeated ED visits for substance use concerns to improve the unmet needs of mental health and addiction service/treatment systems [28]. Existing studies on factors associated with substance use-related ED visits are limited and mostly focus on alcohol and cannabis use [20–22]. Accordingly, the present study adds to the existing literature by (1) using a provincial, population-based database of ED presentations among adolescents and young adults in Ontario, Canada; (2) investigating patient and hospital characteristics associated with repeated substance use-related ED visits; and (3) studying the patterns of use of a wide variety of psychoactive substances, including alcohol, cannabis, opioids, cocaine, stimulants, sedatives, and multiple psychoactive substances. Establishing a pre-COVID-19 trend for substance use-related ED visits may serve as a basis to effectively plan for ED services in the pandemic recovery period.

## Methods

### Study design and data source

A population-based, repeated cross-sectional study examining trends (2008, 2013, and 2018) of repeated ED visits for substance use among adolescents and young adults (aged 13 to 25 years) in the province of Ontario, Canada, was conducted. The study used mental health and substance use-related ED visit data from the National Ambulatory Care Reporting System (NACRS). NACRS contains data for all hospital-based and community-based ambulatory care, including day surgery, outpatient and community-based clinics, and emergency departments. The study used the International Classification of Diseases, 10th Revision (ICD-10) to identify the reason for the ED visit as well as the substance(s). Other variables extracted from NACRS were patient characteristics (age, sex) and hospital characteristics (hospital size, urbanicity, triage level, waiting time). For repeated ED patients, wait-time and triage levels were taken from their first visit. Any raw data recorded as unknown or unavailable (N = 496) were treated as missing data and were removed. The final sample for analyses included 41,121 adolescents and young adults (mean age = 19.46, SD = 3.33, male = 54.8%). The Waypoint Centre for Mental Healthcare ethics review board determined that the study was exempt from ethics approval as the study used de-identified and compiled provincial population data (NACRS) according to Tri-Council Policy Statement (TCPS) article 2.4.

### Study variables

#### Patient characteristics

Age was grouped as either adolescents (13 to 18 years) or young adults (19 to 25 years) to enable comparison between the age groups. Sex was categorized as female or male.

#### Substance use

The class of psychoactive substances consumed or deemed responsible for the poisoning by youth during their ED visits was extracted from the ICD-10 code collected in NACRS dataset (see S1 Table for details).

#### Urbanicity

Urbanicity was defined as the population density of each hospital’s location and was categorized into urban and rural. A rural hospital was defined as a hospital located in an area with fewer than 29,999 inhabitants and a population density of less than 400/km^2^. An urban hospital was defined as a hospital located in an area with a population that is 30,000 inhabitants or greater and a population density that is 400/km^2^ or greater. Categorization was based on the population centers and rural area classification in 2016, which is the current departmental standard in Canada.

#### Hospital size

Hospital size was coded into three categories (small = 0, anything less than 200 beds; medium = 1, 200–400 beds; large = 2, greater than 400 beds).

#### Triage level

Triage level refers to the type and severity of the patient’s initial presenting signs and symptoms and is based on the Canadian Triage and Acuity Scale [29]. The triage level was coded into three categories; those triaged with a level of non-urgent or less-urgent/semi-urgent were coded as "less-urgent," those triaged as urgent/potentially serious were coded as "urgent," and those triaged as emergent/potentially life-threatening and resuscitation/life-threatening were coded as "emergent."

#### Waiting time

Waiting time refers to the duration of time (hours) waiting in the ED from initial presentation until the first assessment by an available physician or other most responsible health care provider. Waiting time was coded into three categories; less than 4 hours, 4–6 hours, and greater than 6 hours.

#### Repeated ED visit status

The frequency of each individual’s ED visit(s) within the fiscal year in the province of Ontario was coded into two categories; one visit (one-time visit), more than one visit in that fiscal year (repeated visit).

#### Year

Three fiscal years (April 1 to March 31) were coded accordingly (i.e. 2008/2009, 2013/2014, 2018/2019).

### Data analysis

To examine the time trend in the repeated ED visits for substance use-related concerns, a bi-variate distribution of patient (age, sex) and hospital characteristics (hospital size, urbanicity, triage level, ED wait time) by repeated ED visit status were analyzed for each year (2008, 2013, 2018). Furthermore, a series of unadjusted binary logistic regression models were conducted to determine the strength of the associations between patient and hospital characteristics and repeated ED visits for each year. Subsequently, adjusted binary regression models were conducted to test the association between hospital-related factors and repeated ED visits controlling for patient characteristics. The adjusted models were conducted for each year (2008, 2013, 2018) and with summed years testing the incremental trend of repeated ED visits over the years. Unadjusted and adjusted odds ratios (ORs) and 95% confidence intervals (CIs) are reported. To understand and compare the magnitude of probabilities of the psychoactive substances used for repeated ED visits, a margins plot was conducted modelling the probability of repeated ED visit status as a function of psychoactive substances used adjusting for patient and hospital characteristics (margins and 95% CI). All analyses were conducted using Software for Statistics and Data Science (STATA; version 16.0) [30].

## Results

In the years assessed (2008, 2013, and 2018), a total of 41,121 adolescents and young adults visited EDs for substance use concerns in the province of Ontario. Table 1 presents the proportion of ED visits (2008, 2013, and 2018) by the patient (age, sex) and hospital characteristics (hospital size, urbanicity, and hospital wait time), triage level, the substance used, and repeated visits. The number of ED visits for substance use among youth consistently increased from 2008 (N = 11,731; 28.50%) to 2013 (N = 13,095; 31.80%) reaching the highest number in 2018 (N = 16,295; 39.6%). The number of repeat visits also increased over the years (2008 N = 1469, 12.52%; 2013 N = 1947, 14.87%; 2018 N = 3291, 20.19%). Further, cannabis-related ED visits have significantly increased from 2008 to 2018 (2008 = 5.35%; 2013 = 7.37%; 2018 = 14.87%).

**Table 1:** Descriptive (N/%) of patient and hospital correlates of substance-related ED visits.

	2008 (N = 11,731; 28.52%)	2013 (N = 13,095; 31.84%)	2018 (N = 16,302; 39.64%)
Age (N, %)			
Adolescents (13–17 yrs.)	3864 (32.94%)	2970 (22.68%)	3307 (20.29%)
Young Adults (18–25 yrs.)	7867 (67.06%)	10125 (77.32%)	12995 (79.71%)
Sex			
Female	5383 (45.89%)	5972 (45.61%)	7252 (44.50%)
Male	6348 (54.11%)	7123 (54.39%)	9043 (55.50%)
Hospital Size (# of beds)			
<200	4058 (34.59%)	4148 (31.68%)	4694 (28.79%)
200–400	3480 (29.66%)	3394 (25.92%)	4621 (28.35%)
<400	4193 (35.74%)	5553 (42.41%)	6987 (42.86%)
Urbanicity			
Rural	2424 (20.66%)	2086 (15.93%)	2691 (16.51%)
Urban	9307 (79.34%)	11009 (84.07%)	13611 (83.49%)
Hospital Wait Time			
< 4 hrs	6035 (52.03%)	6930 (53.02%)	8333(51.26%)
4–6 hrs	2559 (22.06%)	2876 (22.00%)	3442 (21.17%)
>6 hours	3006 (25.91%)	3264 (24.97%)	4482 (27.57%)
Triage Level			
Less urgent	2220 (18.94%)	1467 (11.24%)	2009 (12.37%)
Urgent	5548 (47.33%)	6086 (46.61%)	7407 (45.60%)
Emergent	3953 (33.73%)	5503 (42.15%)	6827 (42.03%)
Substance Use			
Alcohol	7192 (64.17%)	7769 (61.89%)	7557 (48.85%)
Multiple psychoactive	1213 (10.82%)	1431 (11.40%)	1856 (11.76%)
Cannabis	600 (5.35%)	925 (7.37%)	2334 (14.87%)
Opioid	800 (7.14%)	913 (7.27%)	1341 (4.88%)
Cocaine	473 (4.22%)	407 (3.24%)	766 (4.88%)
Stimulant	313 (2.79%)	425 (3.39%)	745 (4.75%)
Sedative	617 (5.50%)	682 (5.43%)	997 (6.35%)
Hospital Visits			
One Visit	10262 (87.48%)	11148 (85.13%)	13011 (79.81%)
Repeat Visit (>1)	1469 (12.52%)	1947(14.87%)	3291 (20.19%)

Table 2 presents the proportion and unadjusted odd-ratios (ORs) and 95% confidence intervals (95% CI) of repeated ED visit status (one-time visit vs. repeated visit) by year, age group, sex, hospital size, urbanity, waiting time and triage level. While the percentage of adolescents’ repeated ED visits for substance use-related conditions decreased over the year assessed (22.1%, 16.3%, and 14.8%, respectively), young adults’ repeated visits increased (77.9%, 83.7% and 85.2%, respectively). Consequently, the unadjusted odds of young adults’ repeated ED visits was 1.60 to 1.86 times higher compared to adolescents. Furthermore, while no significant sex difference was found in the year 2008, males were significantly more likely to re-visit ED than females in the year 2013 [1.21 (1.10–1.33)] and 2018 [1.15 (1.06–1.24)]. Medium-sized hospitals (200–400 beds) were consistently more positively associated with repeated ED visits than were small-sized hospitals (<200 beds). Hospitals in urban areas were significantly and negatively associated with repeat ED visitors than in rural areas in the year 2013 [0.84 (0.74–0.96)] and 2018 [0.90 (0.81–1.00)] but not in 2008. Longer wait-time (>6 hours) was also positively associated with repeated ED visits in the year 2013 [1.19 (1.06–1.33)] and 2018 [1.52 (1.40–1.66)]. Lastly, except for the year 2018, repeated ED visitors were significantly and negatively associated with more severe triage levels. For instance, in the year 2008, urgent and emergent triage levels reduced the odds of repeat visits by 0.84–0.65 times than less urgent triage levels.

**Table 2:** Proportion (%) and unadjusted associations (OR, 95% CI) of repeated ED visits for substance use presentation in 2008, 2013, and 2018.

	2008			2013			2018		
	1 visit (%)	>1 visits (%)	OR (95% CI)	1 visit (%)	>1 visits (%)	OR (95% CI)	1 visit (%)	>1 visits (%)	OR (95% CI)
Age									
Adolescents	34.50	22.06	Ref	23.80	16.28	Ref	21.68	14.77	Ref
Young adults	65.50	77.94	1.86 (1.63–2.12)***	76.20	83.72	1.61 (1.41–1.83)***	78.32	85.23	1.60 (1.44–1.77)***
Sex									
Female	45.80	46.49	Ref	46.30	41.65	Ref	45.19	41.80	Ref
Male	54.20	53.51	0.97 (0.87–1.09)	53.70	58.35	1.21 (1.10–1.33)***	54.81	58.20	1.15 (1.06–1.24)***
Hospital size									
Small (<200 beds)	34.83	32.95	Ref	31.51	32.61	Ref	28.78	28.87	Ref
Medium (200–400 beds)	29.24	32.61	1.18 (1.03–1.35)*	25.26	29.69	1.14 (1.00–1.28)*	27.72	30.81	1.11 (1.00–1.22)*
Large (>600 beds)	35.93	34.45	1.01 (0.89–1.16)	43.23	37.70	0.84 (0.75–0.95)**	43.50	40.32	0.92 (0.84–1.01)
Urbanicity									
Rural	20.49	21.85	Ref	15.57	17.98	Ref	16.21	17.68	Ref
Urban	79.51	78.15	0.92 (0.81–1.05)	84.43	82.02	0.84 (0.74–0.96)**	83.79	82.32	0.90 (0.81–1.00)*
Waiting time									
< 4hrs	51.73	54.09	Ref	53.16	52.25	Ref	52.33	47.00	Ref
4–6 hrs	22.53	18.77	0.80 (0.69–0.92)**	22.48	19.28	0.87 (0.77–0.99)*	21.99	17.93	0.91 (0.82–1.01)
>6 hrs	25.74	27.15	1.01 (0.89–1.15)	24.36	28.48	1.19 (1.06–1.33)**	25.67	35.07	1.52 (1.40–1.66)***
Triage Level									
Less urgent	18.34	23.12	Ref	10.62	14.80	Ref	12.27	12.76	Ref
Urgent	47.14	48.70	0.82 (0.71–0.94)**	46.86	45.19	0.69 (0.60–0.80)***	45.64	45.44	0.96 (0.85–1.08)
Emergent	34.52	28.17	0.65 (0.55–0.76)***	42.52	40.01	0.67 (0.58–0.78)***	42.09	41.80	0.95 (0.84–1.08)

* < .05,

** < .01,

*** < .001;

OR = Odd Ratio, CI = Confidence interval

Table 3 presents the adjusted odds ratios (ORs) and 95% confidence intervals (95% CI) for the association between age, sex, hospital size, urbanicity, wait-time and triage level and repeated ED visits for substance use presentations in 2008, 2013, and 2018. An overall model with adjusted ORs and 95% CI for the associations in the combined years 2008, 2013, and 2018 as a separate factor is also presented in Table 3. The pattern and strength of the associations remained similar in the fully adjusted models. For instance, in the overall model, factors such as young adults, males, medium-sized hospitals, longer than 6 hours of wait time and year were significantly and positively associated with repeated ED visits. At the same time, triage level and urbanicity were significantly and negatively associated with repeated ED visits. Additionally, the adjusted model demonstrates increased odds for repeated ED visits (compared to one ED visit) in 2013 (OR = 1.21) and 2018 (OR = 1.72) compared to 2008.

**Table 3:** Associations between triage level and hospital-related factors and repeated visits in the year 2008, 2013, and 2018 (OR, 95% CI).

	2008	2013	2018	Total
Age				
Adolescents	Ref	Ref	Ref	Ref
Young adults	1.84 (1.61–2.11)***	1.58 (1.39–1.80)***	1.63 (1.47–1.82)***	1.68 (1.57–1.80)***
Sex				
Female	Ref	Ref	Ref	Ref
Male	0.93 (0.83–1.04)	1.17 (1.06–1.29)**	1.13 (1.05–1.23)**	1.09 (1.03–1.15)**
Hospital size				
Small (<200 beds)	Ref	Ref	Ref	Ref
Medium (200–400 beds)	1.25 (1.07–1.48)**	1.22 (1.05–1.42)**	1.15 (1.01–1.30)*	1.20 (1.10–1.30)***
Large (>600 beds)	1.06 (0.90–1.26)	0.91 (0.79–1.05)	0.95 (0.84–1.08)	0.96 (0.88–1.04)
Urbanicity				
Rural	Ref	Ref	Ref	Ref
Urban	0.85 (0.71–1.00)	0.86 (0.73–1.02)	0.86 (0.75–0.99)*	0.86 (0.78–0.94)**
Waiting time				
< 4hrs	Ref	Ref	Ref	Ref
4–6 hrs	0.86 (0.74–0.99)*	0.93 (0.82–1.06)	0.93 (0.83–1.03)	0.91 (0.85–0.98)*
>6 hrs	1.14 (1.00–1.31)	1.30 (1.15–1.46)***	1.56 (1.42–1.71)***	1.38 (1.29–1.47)***
Triage Level				
Less urgent	Ref	Ref	Ref	Ref
Urgent	0.87 (0.75–1.00)	0.72 (0.62–0.83)***	0.94 (0.83–1.07)	0.84 (0.78–0.91)***
Emergent	0.65 (0.58–0.81)***	0.67 (0.57–0.79)***	0.88 (0.77–1.00)*	0.76 (0.70–0.83)***
Year				
2008	N/A	N/A	N/A	Ref
2013				1.21 (1.13–1.31)***
2018				1.72 (1.60–1.84)***

* < .05,

** < .01,

*** < .001

**Fig 1:**
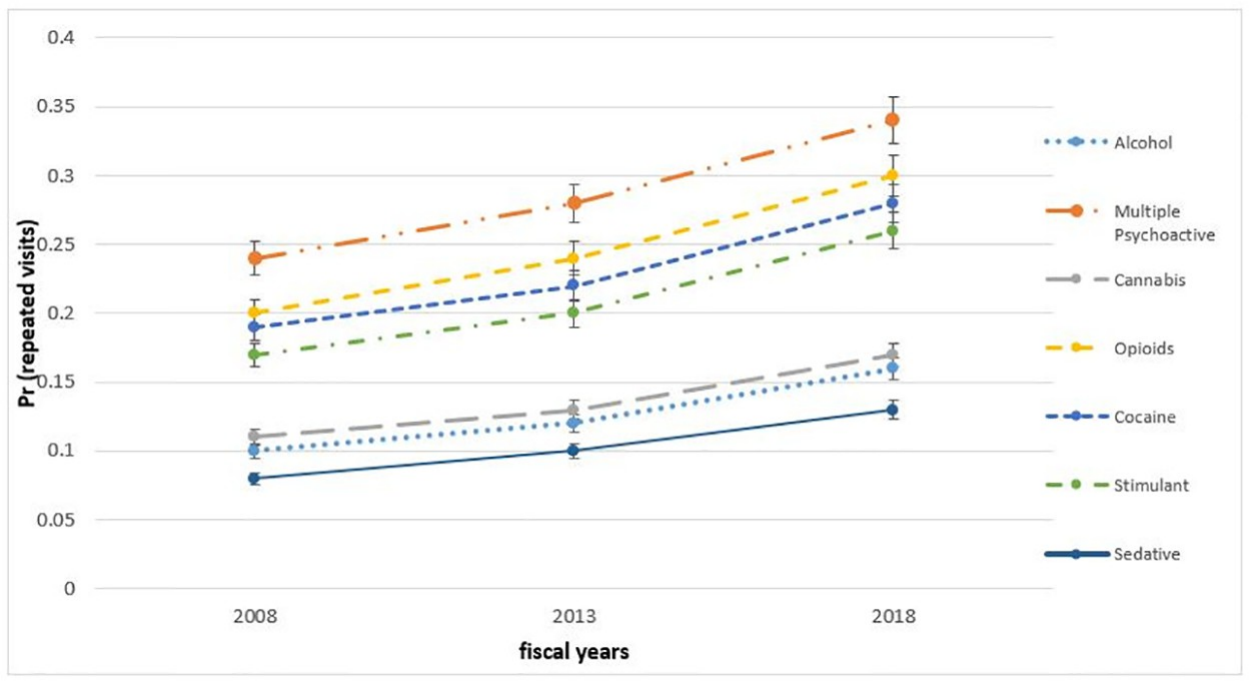
Plot for the adjusted predictions (coefficients; Y-axis) and 95% CI for repeated visits stratified by substances in the years 2008, 2013, and 2018 (X-axis).

Fig 1 depicts the margins and 95% CI, predicting the probabilities of each class of substance consumed for repeated ED visits while adjusting for other variables (i.e., age, sex, hospital size, urbanicity, wait time and triage level; see S2 Table). Holding all other factors constant, using multiple psychoactive substances most strongly predicted repeated ED visits, followed by opioids, cocaine and stimulants. Youth who consumed cannabis, alcohol and sedatives were less likely to re-visit ED.

## Discussion

The proportion of substance use-related repeated ED visits significantly and consistently increased in 2013 and 2018 compared to 2008. Young adults, males, medium-size hospitals, rural locations, wait times longer than 6 hours, and symptom severity was significantly associated with the repeated ED visits. Further, polysubstance use and specific psychoactive substances such as opioids, cocaine, and stimulants were strongly associated with repeated ED visits compared to substances such as cannabis, alcohol and sedatives.

The current findings confirm the increasing trend of ED visits and repeated ED visits over time among Canadian youth for substance use presentations [6, 13, 14]. Specifically, while the proportion of alcohol-related ED visits remained relatively constant in the last decade, a substantial increase in cannabis- and opioid-related ED visits was evident in the current study [21, 22, 24]. The widespread availability of unregulated cannabis products in the time preceeding legalization (fall of 2018) of non-medical cannabis use in Canada may have contributed to this substantial increase in cannabis-related ED visits during 2018 [31]. Further, although alcohol-related ED visits have been relatively constant in the last decade, alcohol intoxication was the most frequent reason that brought youth to the ED over the years, [20, 32]. Providing targeted resources by trained healthcare professionals for substance use problems to this population is warranted [1, 13, 14].

Heroin and synthetic opioid poisoning also largely accounted for the increased ED visits in the last decade. For example, polysubstance and opioids were the substances that most strongly predicted repeated ED visits. A recent study reports a 4 to 10 fold increase in synthetic opioid-related ED visits across Canada from 2007–2008 to 2016–2017 [24]. Identifying the type of psychoactive substances that bring youth to the ED and, more importantly, incur repeated visits is critical to prioritize the targeted intervention efforts and continue monitoring and evaluating the impact of specific interventions and related harms among young people. Returning to the ED after an initial visit for opioid, cocaine, stimulant, or cannabis consumption may indicate that follow-up care, including the referrals for services and supports, was not available/accessible for those initial ED patients, pointing to gaps in the health care system. Providing effective services and supports to youth in the community outside the ED is critical.

The present study also investigated patient and hospital factors contributing to repeated ED visits in the past decade among youth. Patient characteristics, such as the young adult age group and being male, significantly and continuously contributed to the increased odds of repeated ED visits, consistent with previous findings [32]. Young adults might consume more psychoactive substances that may lead to ED than adolescents due to the substance-use involving cultural norms that young adults encounter in post-secondary education settings and the easier access to legalized psychoactive substances, such as alcohol and cannabis.

Hospital characteristics, such as medium-size hospitals, were also associated with repeated ED visits. While large-sized hospitals were not linked with repeated ED visits, hospitals in rural areas were significantly associated with repeated ED visits. These findings resonate with previous studies that found that patients who visit the ED frequently for mental health and addiction concerns are more likely to live in lower-income neighbourhoods than in higher-income neighbourhoods [6, 10]. Similarly, youth in rural areas are significantly more likely to consume alcohol and to engage in high-risk drinking behaviours (e.g., binging/getting intoxicated) [33], which may in part explain the findings that rural hospitals have increased repeat ED visits. Additionally, access to mental health specialists for substance use problems is not equitable across the province [6] in Canada. Specifically, larger-sized hospitals in more urban areas have more specialized services like withdrawal management centers (www.connexontario.ca). Withdrawal management centers often have specialist addiction workers who support connecting the patients to community treatment resources. We speculate that larger, urban-based health centers maintain, or have access to, a broader range of support services and other resources. As a result, ED presenting MHA challenges is more likely to be addressed, thereby lessening repeat visits.

The present study also identified an alarming increase in cases of young adults with Emergent triage levels between 2008–2018, which seems unrelated with the opioid, cocaine or polysubstance exposure. We speculate that perhaps increasing unpredictability in the constituents of drugs bought and sold in the unregulated, illegal market drugs may account for the increase in emergent ED presentations [34]. For example, fentanyl is increasingly being found in unpredictable quantities in opioid and non-opioid street drugs [35, 36]. Further, in the lead up to legalization of cannabis in Canada, concentrations of Δ-9-tetrahydrocannabiol (THC) in some cannabis productss had increased [37]. Compared to low-potency cannabis, high-potency cannabis is associated with a greater risk of psychotic symptoms [37]. Synthetic cannabinoids have also been detected in the unregulated drug market, which are associated with more serious adverse events [37] Lastly, our findings indicate that substance-related ED patients who received higher triage scores (i.e. more severe symptoms) in their admission were more likely to meet physicians faster than those triaged lower, which is an expected finding. Also, those repeated ED patients for substance use-related problems tended to wait longer to be seen in the ED. Patients with mental health and addiction concerns do not necessarily receive lower triage scores or wait longer than other patients in ED [38]. However, long wait time in EDs can incur serious health consequences, and be associated with patient dissatisfaction, a higher rate of patients leaving without being seen, and a longer length of stay [39, 40]. The American College of Emergency Physicians identified that active bed management (i.e., reducing bed occupancy for patients waiting to leave) is crucial to reduce ED wait time [40]. Further, a reverse triage system (i.e., ranking patients by risk for an adverse event at the end of ED care and discharging those at low risk) can also be adopted to reduce ED wait time [40] and subsequently reduce the number of young people visiting ED for substance use problems repeatedly.

There are several limitations to this current study. The cross-sectional design precludes our ability to understand the temporal ordering between patient and hospital characteristics and repeated ED visits. Furthermore, our findings do not reflect the most up-to-date trend of cannabis-related ED visits post-legalization of non-medical cannabis use in Canada. Lastly, as a secondary analysis of an existing dataset, other factors (e.g. disposition, treatment received, other medical diagnoses, death during ED visit or following ED visit, and follow-ups) that may be associated with repeated ED vists were not considered in the present analysis.

Despite the limitations, the current study uniquely addresses the lack of existing literature on evidence-based factors contributing to repeated ED visits for substance use concerns among adolescents and young adults. It informs current policy and practices for improving the mental health and addiction care system. For example, understanding pre-pandemic trends of substance use-related ED visits can help determine workforce planning and development needs in light of the post-pandemic shift. Current findings highlight several practical implications. First, the existing fast-track mental healthcare clinics (e.g., Rapid Access Addiction Medicine; RAAM) and telehealth are critical to diverting non-emergent substance-related ED patients and providing efficient care to reduce repeated substance-related ED visits. Second, the fast-track mental healthcare clinics/telehealth should make special efforts to engage and develop specific (e.g., withdrawal management) programming for repeated ED visitors for the classes of psychoactive substances identified in the current study. Most importantly, fast-track mental healthcare clinics/telehealth should be evenly distributed and accessible across the province, especially in smaller-sized hospitals and rural areas.

## Supporting information

S1 Table ICD-10 code inclusion. (DOCX)

S2 Table Associations between triage level, hospital-related factors, and psychoactive substance and repeated visits in the year 2008, 2013, and 2018 (OR, 95% CI). * < .05, ** < .01, *** < .001.
(DOCX)
