# Supplementary material for: Trends of repeated emergency department visits among adolescents and young adults for substance use: A repeated cross-sectional study
Source: PLoS One. 2023 Feb 22;18(2):e0282056. doi: 10.1371/journal.pone.0282056 (PMC9946266; doi:10.1371/journal.pone.0282056)
Supplement: S1 Table — (DOCX) [file pone.0282056.s001.docx]

S1 Table. ICD-10 code inclusion

| **ED Visit Category** | **ICD-10 Codes** |
| --- | --- |
| Mental and behavioural disorders due to use of psychoactive substances (F100-199) | F100-F109, F110-F116, F118, F119, F120-F129, F130-F135, F138, F139, F140-F143, F145, F147-F149, F150-F153, F155, F156, F158, F159, F160-F163, F165, F166, F168, F169, F170-F173, F180-F183, F185, F186, F189, F190-F199 |
| Finding psychoactive substance in the blood (R 700-799) | R780, R781, R784-R786, R788, R789 |
| Poisoning (T 400-499) | T400-T402, T4020-T4023, T4028, T403, T404, T4040, T4041, T4048, T405-T412, T420-T424, T426, T427, T436, T438, T439 |
| Toxic effect (T 500-699) | T510-T512, T518-T520, T522-T524, T528, T529, T531, T534-T537, T539, T652, T658, T659 |
