# Supplementary material for: Trends of repeated emergency department visits among adolescents and young adults for substance use: A repeated cross-sectional study
Source: PLoS One. 2023 Feb 22;18(2):e0282056. doi: 10.1371/journal.pone.0282056 (PMC9946266; doi:10.1371/journal.pone.0282056)
Supplement: S2 Table — * < .05, ** < .01, *** < .001. (DOCX) [file pone.0282056.s002.docx]

S2 Table. Associations between triage level, hospital-related factors, and psychoactive substance and repeated visits in the year 2008, 2013, and 2018 (OR, 95% CI)

|  | Repeat Visits |
| --- | --- |
|  | OR (95% CI) |
| Age |  |
| Adolescents | Ref |
| Young Adults | 1.53 (1.42-1.64)*** |
| Sex |  |
| Female | Ref |
| Male | 1.04 (0.98-1.10) |
| Hospital Size |  |
| Small (<200 beds) | Ref |
| Medium (200-400 beds) | 1.20 (1.10-1.30)*** |
| Large (>400 beds) | 0.99 (0.91-1.08) |
| Urbanity |  |
| Rural | Ref |
| Urban | 0.84 (0.76-0.92)*** |
| Waiting Time |  |
| < 4hrs | Ref |
| 4-6 hrs | 0.94 (0.87-1.01) |
| > 6 hrs | 1.42 (1.33-1.52)*** |
| Triage Level |  |
| Less urgent | Ref |
| Urgent | 0.86 (0.79-0.94)*** |
| Emergent | 0.77 (0.71-0.84)*** |
| Year |  |
| 2008 | Ref |
| 2013 | 1.22 (1.13-1.32)*** |
| 2018 | 1.69 (1.57-1.81)*** |
| Substance |  |
| Alcohol | Ref |
| Multiple Psychoactive | 2.82 (2.61-3.04)*** |
| Cannabis | 1.12 (1.01-1.23)* |
| Opioids | 2.27 (2.07-2.49)*** |
| Cocaine | 2.10 (1.87-2.37)*** |
| Stimulant | 1.88 (1.65-2.14)*** |
| Sedative | 0.77 (0.67-0.89)*** |

* < .05, ** < .01, *** < .001
